# Characterizing Long-Term Patterns of Weight Change in China Using Latent Class Trajectory Modeling

**DOI:** 10.1371/journal.pone.0116190

**Published:** 2015-02-20

**Authors:** Lauren Paynter, Elizabeth Koehler, Annie Green Howard, Amy H. Herring, Penny Gordon-Larsen

**Affiliations:** 1 Department of Nutrition, Gillings School of Global Public Health at the University of North Carolina, Chapel Hill NC, United States of America; 2 Department of Biostatistics, Gillings School of Global Public Health at the University of North Carolina, Chapel Hill NC, United States of America; 3 Carolina Population Center, University of North Carolina, Chapel Hill NC, United States of America; National Institute of Agronomic Research, FRANCE

## Abstract

**Background:**

Over the past three decades, obesity-related diseases have increased tremendously in China, and are now the leading causes of morbidity and mortality. Patterns of weight change can be used to predict risk of obesity-related diseases, increase understanding of etiology of disease risk, identify groups at particularly high risk, and shape prevention strategies.

**Methods:**

Latent class trajectory modeling was used to compute weight change trajectories for adults aged 18 to 66 using the China Health and Nutrition Survey (CHNS) data (n = 12,611). Weight change trajectories were computed separately for males and females by age group at baseline due to differential age-related patterns of weight gain in China with urbanization. Generalized linear mixed effects models examined the association between weight change trajectories and baseline characteristics including urbanicity, BMI category, age, and year of study entry.

**Results:**

Trajectory classes were identified for each of six age-sex subgroups corresponding to various degrees of weight loss, maintenance and weight gain. Baseline BMI status was a significant predictor of trajectory membership for all age-sex subgroups. Baseline overweight/obesity increased odds of following ‘initial loss with maintenance’ trajectories. We found no significant association between baseline urbanization and trajectory membership after controlling for other covariates.

**Conclusion:**

Trajectory analysis identified patterns of weight change for age by gender groups. Lack of association between baseline urbanization status and trajectory membership suggests that living in a rural environment at baseline was not protective. Analyses identified age-specific nuances in weight change patterns, pointing to the importance of subgroup analyses in future research.

## Introduction

While obesity had been considered a result of a modern lifestyle, obesity is a growing public health challenge in both modern and developing countries [1]. With modernization over the past three decades, obesity has increased tremendously in China [2]. This trend towards increasing weight has also led to high rates of obesity-related non-communicable diseases such that these diseases are the leading causes of morbidity, disability and mortality [3].

Given the association of obesity and weight gain with chronic disease risk, it is important to identify population subsets at highest risk in order to intervene appropriately to reduce mortality and morbidity. Identification of different patterns of weight change may provide a useful tool for detecting within-population groups at increased risk of chronic disease, and allow for introduction of strategic public health interventions which may help reduce the magnitude of chronic disease in targeted populations [4, 5].

Latent class trajectory modeling is one such method of identifying distinct groups with similar underlying trajectories in longitudinal data [6, 7]. Longitudinal studies can be challenging to summarize due to the magnitude of data provided by long term studies. Multivariate analysis of variance (MANOVA) and structural equation modeling (SEM) are able to estimate growth trajectories over time; however, these methods produce an average trajectory for an entire population and may not be appropriate in settings with more heterogeneous populations [6]. While repeated measures analysis of variance (ANOVA) and analysis of covariance (ANCOVA) allow individual-specific growth trajectories, they do not facilitate straightforward identification of distinct groups of individuals. Latent class analysis allows researchers to summarize data across multiple time points in an unbiased manner to identify patterns because this method does not require a priori knowledge about the number or direction of existing trajectories in a given population [5, 6]. Thus, latent class analysis is a useful tool for summarizing data to identify high risk groups that can then be targeted for intervention or prevention strategies.

In this paper we take advantage of 18 years of longitudinal weight data on 12,611 individuals (48,629 observations) where anthropometric data were collected by trained health care workers [3]. Data were used to derive trajectory patterns of weight change and examine correlates of such patterns. While this method has been applied to research questions in the fields of psychology, sociology, and criminology focusing on behavioral and physical development trajectories for children and adolescents [8–12], few studies have applied latent class trajectory methods to study weight change in populations undergoing modernization with rapid weight change. Other research has computed BMI trajectories for children, focusing on identifying prevalence of overweight and obesity over time rather than identification of patterns of weight change [13]. Additionally, while there are other published studies spanning long periods of follow up, the majority of this existing research is based on self-reported (rather than measured) height and weight data [14–17], in contrast to our data which are based on measured anthropometry. We hypothesize that patterns of increasing weight gain are more prevalent in individuals who, at baseline, are 1) living in more (versus less) urban areas and 2) are overweight or obese. We also hypothesize that these weight trajectory patterns will differ by age and gender subgroups.

## Methods

### Study Population

Data were from the China Health and Nutrition Survey (CHNS), a large-scale household-based, longitudinal survey in China. The CHNS collected health data in 228 communities in nine diverse provinces (Guangxi, Guizhou, Heilongjian, Henan, Hubei, Hunan, Jiangsu, Liaoning, and Shandong) throughout China from 1989–2009 with eight rounds of surveys. Using multistage, cluster sampling, two cities (one large and one small city—usually the provincial capital and a lower income city) and four counties (stratified by income: one high, one low, and two middle income counties—for a total of four counties per province) were selected. Villages and small towns within counties and urban and suburban neighborhoods within cities were selected as defined politically and geographically based on State Statistical Office definitions. Twenty households per community were then selected for participation. The surveyed provinces represent 56% of the Chinese population. The CHNS sample is not representative of China, although the sample is designed to obtain a variety of economic and demographic circumstances. The study was approved by the Institutional Review Board at the University of North Carolina at Chapel Hill, the China–Japan Friendship Hospital, the Ministry of Health and China, and the Institute of Nutrition and Food Safety, China Centers for Disease Control. Participants gave informed consent and data are housed at the Carolina Population Center at the University of Carolina. Survey procedures have been described elsewhere [18].

### Analysis sample

Our sample included eight waves of data from 1991 through 2009 (1991, 1993, 1997, 2000, 2004, 2006, 2009), with variation in timing of study entry due to design (family formation, childbirth, and addition of replacement communities result in some variation in timing of study entry). Analyses were limited to adults, defined as 18 years or older at baseline measurement and less than 66 years of age with at least two survey visits with anthropometric measurements (n = 12,611; the proportion of the analytic sample with complete data by number of repeat visits is presented in S1 Table). If a woman reported being pregnant during a particular survey year, her weight is set to missing to ensure that any changes to weight gain during this time period did not skew the results.

### Anthropometry

Weight and height measures were collected at each survey by trained health workers who followed standard protocol and techniques. Weight was measured in light indoor clothing without shoes to the nearest tenth of a kilogram with a beam balance scale. Height was measured without shoes to the nearest tenth of a centimeter with a portable stadiometer [3]. Weight change was calculated as current weight minus baseline weight.

Given our focus on adults, we assume no change in height throughout the follow-up and thus use a single adult height measurement derived from average height to calculate baseline BMI (weight in kg)/height in m^2^). We classified underweight, normal weight, overweight/obese according to Asian BMI cut points due to the significant mortality risks that are associated with lower BMI values in this population (<18.5 kg/m^2^, 18.5–22.9 kg/m^2^, and > = 23.0 kg/m^2^ respectively) [19, 20].

### Covariates

Year of study entry is included as an individual’s baseline time indicator. Baseline age was examined as a continuous variable scaled by a factor of 10 such that interpretation of corresponding odds ratios was in terms of risk associated with a 10 year increase in age. A dichotomous urbanization variable represented whether an individual’s household was in an urban or rural setting at baseline, as defined by the Chinese government.

### Statistical Analysis

Given the dramatic secular trends in weight gain in China over the past 18 years as well as strong sex and cohort effects related to urbanization [21], there was conceptual rationale to examine trajectories within age groups [22]. This is in contrast to other approaches, such as Ostbye et al. [14] to derive overall trajectories, given the consistency of trajectories by age and sex in the US context [14]. In our case, we expected patterns in weight trajectories to vary by age and sex, due to urbanization-related changes [21]. We used latent class trajectory analysis (LCTA) to identify trajectories [10].

Analyses were conducted using SAS version 9.3 (SAS Institute, Cary, NC). Identification of weight change trajectories is through latent class trajectory modeling with the TRAJ procedure using the censored normal model [7, 10]. We modeled weight change over time for six baseline age by gender subgroups corresponding to the following age categories: 18 to <30, 30 to <40, and 40 to <66 for males and females. For each age by gender subgroup, latent class trajectory models were used to identify weight change patterns, allowing for a variety of different order polynomials in time to determine the best fitting polynomial form. Any group requiring a flexible polynomial was modeled as such, thus we relied on a conservative approach and tailored response. We evaluated the model fit of each trajectory group separately rather than using a single overall structure. In each case, the BIC of the gender-age specific model was superior.

To be eligible for inclusion in trajectory derivation analyses, an individual was required to have a minimum of three weight measurements (two weight change measurements), which included 8,798 individuals. This allowed us to capture the most accurate weight trajectory classes. Next, we assigned the remaining 3,813 individuals having exactly two weight measurements to a trajectory using the posterior predictive probabilities based on the parameter estimates from these models, resulting in our final analysis sample of n = 12,611.

### Criteria for Establishing Trajectory Membership

Within each age and gender strata, all trajectory classes were assumed to follow the same order polynomial, as required by the software, with different models pertaining to linear, quadratic, cubic, fourth, and fifth order polynomials. The best fitting models were chosen based on the following combined criteria: i) lowest BIC with ii) at least 2% predicted sample size in each trajectory class. Some authors have suggested 5% group membership as a criterion for model selection; however, it has been noted that in some cases, derived trajectories may include only a small fraction of individuals in the sample [6]. In the interest of identifying unique patterns and due to our considerable sample size, the less restrictive 2% group membership criterion is used for this paper. It is important to note that the 2% membership refers to predicted group membership produced from model results. Actual group membership may in fact be less than 2%.

### Secondary analyses to examine correlates of weight trajectories

After determining weight change trajectories, the nominal weight trajectory class was used as the outcome variable to examine baseline predictors of weight trajectory class membership using a multinomial generalized linear mixed effects hierarchical model with random intercepts to account for potential community-level correlation using the GLIMMIX procedure in SAS. Included covariates corresponded to baseline measures determined a priori to be of interest: baseline age scaled by a factor of 10 (continuous), year of study entry (continuous), baseline BMI status as a three category variable (underweight, normal weight [referent], overweight/obese), and a dichotomous baseline urban-rural variable (referent: urban). Reference groups for weight change trajectories were chosen on the basis of a pattern indicating “minimal weight change.”

## Results

### Descriptive Statistics

Approximately two-thirds of the sample lived in rural areas at baseline (Table 1). Overweight/obesity was comparatively higher in the older age groups (Table 1; overweight/obesity by survey year is presented in S2 Table).

**Table 1:** Descriptive Statistics for Baseline Covariates by Gender and Age-Group, China Health and Nutrition Survey.

	Gender							
	Male				Female			
	Baseline Age Group				Baseline Age Group			
	18–30	30–40	40–66	**Total**	18–30	30–40	40–66	**Total**
**N = 12,611**	2,225	1,571	2,403	6,199	2,184	1,785	2,443	6,412
**Baseline Age, Mean(Std)**	23.46 (3.67)	35.22 (2.75)	50.16 (6.79)	36.79 (12.58)	24.53 (3.54)	35.03 (2.85)	50.26 (6.68)	37.26 (12.02)
**Baseline Weight(kg), Mean(Std)**	59.53 (8.68)	62.27 (9.79)	61.40 (10.31)	60.95 (9.68)	52.19 (7.11)	54.51 (8.26)	54.96 (9.92)	53.89 (8.67)
**Average Height(cm), Mean(Std)**	168.08 (6.04)	167.14 (6.30)	165.26 (6.33)	166.75 (6.34)	157.09 (5.36)	156.46 (5.70)	154.15 (6.08)	155.79 (5.88)
Baseline BMI Category, %*								
Underweight	11.87	5.92	6.70	8.36	12.00	6.22	7.25	8.58
Normal Weight	69.98	59.96	55.81	61.95	67.12	59.05	45.48	56.63
Overweight/Obese	17.30	33.61	36.75	28.97	20.01	34.57	46.42	34.12
**Urbanicity At Baseline, %**								
Urban	30.92	33.80	36.58	33.84	32.83	33.22	38.89	35.25
Rural	69.08	66.20	63.42	66.16	67.17	66.78	61.11	64.75
Baseline Wave, %**								
1991 (n = 6,711)	44.99	55.57	57.26	52.43	45.33	56.25	60.05	53.98
1993 (n = 857)	11.33	6.24	5.74	7.87	9.89	4.09	3.27	5.75
1997 (n = 2,185)	21.30	17.12	15.19	17.87	18.73	16.25	15.47	16.80
2000 (n = 1,295)	10.56	9.99	8.95	9.79	12.73	10.31	9.25	10.73
2004 (n = 1,098)	7.82	7.96	9.36	8.45	9.43	8.80	8.64	8.95
2006 (n = 465) ***	4.00	3.12	3.50	3.58	3.89	4.31	3.32	3.79

*Overweight and obesity classified using the Asian cut-point (BMI ≥ 23 kg/m^2^) and underweight classified as BMI<18.5 kg/m^2^[20]

**Wave at study entry varies by design of this household-based survey, with study entry due to family formation and childbirth and due to exogenous weather shocks and subsequent replacement enrollment of new villages with identical sampling techniques.

***Eligibility for inclusion in the analysis sample was based on each individual having data from at least two anthropometric visits, thus the latest year for study entry was 2006

### Trajectories

**Fig 1:**
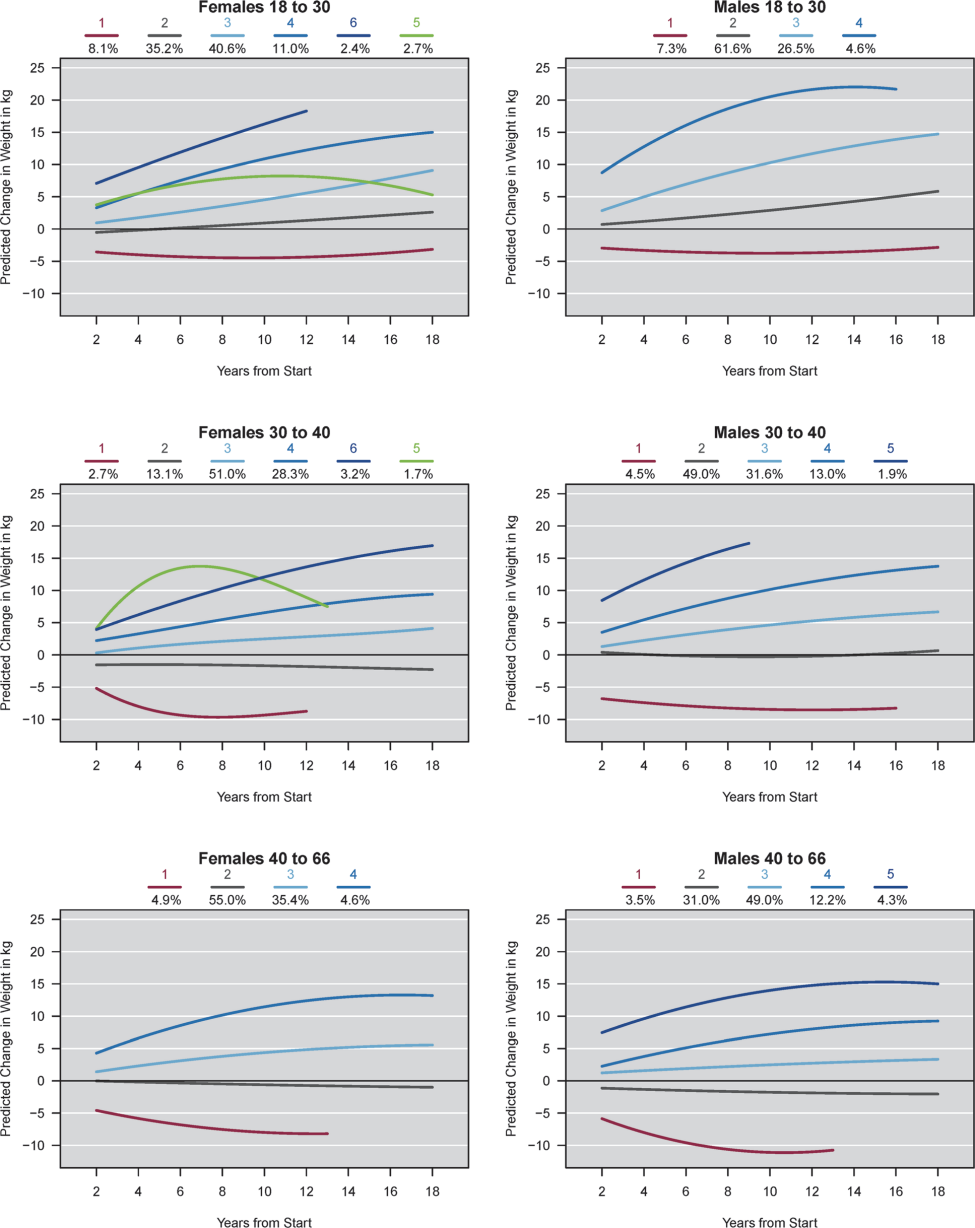
Derived weight trajectories by sex, across three age strata, China Health and Nutrition Survey. Derived weight trajectories are for the 18 year study period. Each age-sex stratum includes a different set of trajectory classes specific to the within-strata weight trajectories. Separate colors indicate each weight trajectory class, with percent of sample in each class for each sex-age strata shown above each figure.

Individuals were assigned to the class with the highest posterior probability (Table 2). In addition, median posterior probabilities and interquartile ranges were computed for each class [6]. Most classes had a high median posterior probability, indicating satisfactory class assignment. Lower medians, as expected, were found in the age-sex subgroups with more classes although these posterior probabilities still suggest satisfactory class assignment relative to random class assignment. Trajectory results for all of the six baseline age-gender analysis groups are shown in Table 2 and Fig. 1.

**Table 2:** Trajectory Descriptions, Median Posterior Probabilities and Group Membership Percentage, China Health and Nutrition Survey*.

Trajectory	N	Median (IQR) Posterior Probability	Description	Percent membership
**Females 18 to 30**	2,184			
Class 1	177	0.81 (0.6,0.97)	Initial Loss with Maintenance	8.1
Class 2	769	0.56 (0.46,0.78)	Maintenance (ref)	35.2
Class 3	887	0.51 (0.41,0.71)	Moderate gain (low)	40.6
Class 4	240	0.59 (0.44,0.89)	Slow gain (medium)	11.0
Class 5	59	0.6 (0.52,0.78)	Initial gain with loss (medium)	2.7
Class 6	52	0.75 (0.54,0.97)	Rapid gain (medium)	2.4
**Females 30 to 40**	1,785			
Class 1	49	0.98 (0.79,1)	Initial Loss with Maintenance	2.7
Class 2	233	0.8 (0.65,0.97)	Maintenance (ref)	13.1
Class 3	910	0.69 (0.53,0.91)	Slow gain (low)	51.0
Class 4	505	0.77 (0.57,0.95)	Moderate gain (medium)	28.3
Class 5	30	0.9 (0.62,0.98)	Initial gain with loss (high)	1.7
Class 6	58	0.95 (0.65,1)	Rapid gain (high)	3.2
**Females 40 to 66**	2,443			
Class 1	120	0.9 (0.68,0.99)	Initial Loss with Maintenance	4.9
Class 2	1344	0.7 (0.57,0.93)	Maintenance (ref)	55.0
Class 3	866	0.78 (0.56,0.95)	Moderate gain (low)	35.4
Class 4	113	0.96 (0.75,1)	Moderate gain (medium)	4.6
**Males 18 to 30**	2,225			
Class 1	163	0.87 (0.67,0.99)	Initial Loss with Maintenance	7.3
Class 2	1370	0.74 (0.61,0.94)	Maintenance and slight gain (ref)	61.6
Class 3	590	0.82 (0.6,0.97)	Moderate gain (medium)	26.5
Class 4	102	0.95 (0.8,1)	Rapid gain (high) with maintenance	4.6
**Males 30 to 40**	1,571			
Class 1	70	0.97 (0.66,1)	Initial Loss with Maintenance	4.5
Class 2	770	0.68 (0.59,0.9)	Maintenance (ref)	49.0
Class 3	497	0.83 (0.63,0.97)	Slow gain (low)	31.6
Class 4	204	0.86 (0.69,0.98)	Moderate gain (medium)	13.0
Class 5	30	0.97 (0.85,1)	Rapid gain (high)	1.9
**Males 40 to 66**	2,403			
Class 1	83	0.9 (0.75,1)	Initial Loss with Maintenance	3.5
Class 2	745	0.69 (0.54,0.88)	Maintenance (ref)	31.0
Class 3	1,177	0.56 (0.49,0.76)	Slow gain (low)	49.0
Class 4	294	0.75 (0.6,0.92)	Moderate gain (medium)	12.2
Class 5	104	0.92 (0.74,0.99)	Rapid gain (high)	4.3

* Weight change trajectories identified through latent class trajectory modeling with the TRAJ procedure using the censored normal model; individuals were assigned to the class with the highest posterior probability.

### Women

The number of weight change trajectories for adult females ranged from four for the baseline 40–66y age group, to six for the 18 to 30y, and 30 to 40y baseline age groups. In the youngest age subgroups for women, the most common weight trajectory was a moderate gain (low) pattern with 40.6% (light blue), and a slow gain (low) with 51.0% (light blue) for females 18 to 30y and 30 to 40y respectively. The majority of the women in the baseline 40–66y age subgroup followed a weight maintenance pattern (55.0%, grey). The youngest baseline age group (18 to 30y) for women had the largest percentage in an ‘initial loss with maintenance’ pattern (red) at 8.1% compared to the older baseline age groups. In the 18 to 30y and 30 to 40y baseline age groups, we identified unique classes of initial weight gain followed by weight loss (2.7%, green and 1.7% respectively, green).

### Men

The number of weight change trajectories was fairly consistent across all analyses for males. Males aged 30 to 40 years and 40 to less than 66 years at baseline had five weight change patterns, while the baseline 18 to 30y had four. The most common pattern for the youngest baseline male age group was a maintenance and slight gain weight trajectory (61.6%, grey) which was also most common for their female counterparts. The most common weight trajectory for the men baseline 30–40y was a maintenance trajectory (49.0%, grey), while for baseline 40-<66y was a slow gain (low) pattern (49.0%, light blue). As for women, the youngest baseline men group had comparatively more males following an ‘initial loss with maintenance’ trajectory (7.3%, red).

The smallest percentages of group membership for both men and women in our analysis sample were seen in the categories with initial weight loss or rapid weight gain.

### Generalized Linear Mixed Effects Models (GLMM)

When considering results for each baseline age by gender subgroup analysis, GLMM models showed that baseline urbanicity was not a significant predictor of trajectory membership after controlling for baseline overweight status, baseline age, and baseline wave (Table 3). However, an exception was seen for young adult males (aged 18–30 years), where baseline urbanicity was significantly associated with membership in the moderate gain (medium) pattern. Young adult males living in a rural area at baseline had lower odds of falling in the moderate weight gain pattern versus the maintenance referent as compared to their urban counterparts, albeit at borderline statistical significance (p = 0.04).

**Table 3:** Odds Ratios and 95% Confidence Intervals for Baseline Covariates Related to Trajectory Membership, China Health and Nutrition Survey.

		Baseline Predictors^†^ of Trajectory Membership^††^: OR (95%CI), CHNS 1991–2009				
Trajectory	N	UW	OW/OB	Urbanicity	Age/10	Year of study entry
**Females 18 to 30**	**2,165**					
Class 1	175	0.22 (0.05, 0.88)*	5.05 (3.37, 7.57)***	1.11 (0.68, 1.79)	0.68 (0.40, 1.14)	1.00 (0.97, 1.04)
Class 2	765					
Class 3	877	1.97 (1.42, 2.73) ***	0.75 (0.58, 0.98) *	0.97 (0.79, 1.20)	1.63 (1.23, 2.15) **	1.00 (0.98, 1.02)
Class 4	239	3.10 (2.02, 4.76) ***	1.00 (0.68, 1.47)	0.92 (0.68, 1.27)	2.95 (1.90, 4.58) ***	0.96 (0.93, 0.99) *
Class 5	59	1.10 (0.39, 3.14)	0.72 (0.32, 1.58)	0.86 (0.34, 2.16)	3.17 (1.32, 7.58) *	0.89 (0.82, 0.96) *
Class 6	50	6.36 (2.97, 13.64) ***	1.11 (0.48, 2.59)	0.66 (0.27, 1.61)	1.48 (0.60, 3.63)	1.03 (0.97, 1.10)
**Females 30 to 40**	**1,782**					
Class 1	49	0.34 (0.00, 228.43)	4.77 (2.11, 10.77)**	0.72 (0.29, 1.79)	0.35 (0.10, 1.17)	1.03 (0.96, 1.11)
Class 2	233					
Class 3	909	3.36 (1.06, 10.68) *	0.53 (0.39, 0.72) ***	0.86 (0.63, 1.18)	0.59 (0.35, 0.99) *	1.02 (0.98, 1.05)
Class 4	503	4.46 (1.38, 14.40) *	0.35 (0.25, 0.49) ***	0.92 (0.64, 1.33)	0.45 (0.25, 0.80) *	0.98 (0.95, 1.02)
Class 5	30	12.85 (2.71, 60.83) *	0.20 (0.06, 0.65) *	1.05 (0.30, 3.74)	0.17 (0.04, 0.85) *	1.01 (0.92, 1.11)
Class 6	58	6.17 (1.37, 27.82) *	0.54 (0.28, 1.07)	1.39 (0.57, 3.37)	0.19 (0.06, 0.60) *	0.90 (0.83, 0.98) *
**Females 40 to 66**	**2,422**					
Class 1	116	0.12 (0.00, 3.57)	4.80 (2.76, 8.35) ***	0.75 (0.43, 1.30)	1.12 (0.82, 1.53)	0.99 (0.95, 1.04)
Class 2	1,334					
Class 3	861	0.95 (0.67, 1.35)	0.53 (0.44, 0.65) ***	0.95 (0.76, 1.18)	0.64 (0.56, 0.74) ***	0.98 (0.96, 0.99) *
Class 4	111	1.36 (0.67, 2.75)	0.42 (0.26, 0.67) **	0.73 (0.40, 1.33)	0.48 (0.34, 0.67) ***	0.97 (0.92, 1.02)
**Males 18 to 30**	**2,206**					
Class 1	159	0.24 (0.08, 0.69) *	4.11 (2.79, 6.04) ***	0.86 (0.55, 1.33)	0.78 (0.48, 1.28)	0.94 (0.90, 0.98) *
Class 2	1,359					
Class 3	588	0.99 (0.73, 1.35)	0.70 (0.52, 0.95) *	0.79 (0.62, 1.01) *	0.81 (0.61, 1.08)	0.99 (0.97, 1.02)
Class 4	100	2.67 (1.50, 4.76) **	1.42 (0.79, 2.56)	0.99 (0.51, 1.91)	0.51 (0.27, 0.97) *	1.05 (1.00, 1.10)
**Males 30 to 40**	**1,563**					
Class 1	69	0.04 (0.00, 21.01)	4.23 (2.34, 7.63) ***	1.14 (0.52, 2.49)	1.43 (0.52, 3.94)	1.02 (0.96, 1.09)
Class 2	764					
Class 3	497	1.08 (0.65, 1.80)	1.63 (1.27, 2.11) **	0.93 (0.71, 1.21)	1.40 (0.91, 2.16)	0.95 (0.93, 0.98) **
Class 4	203	1.16 (0.62, 2.18)	0.58 (0.39, 0.87) *	1.19 (0.71, 1.67)	0.50 (0.27, 0.93) *	0.98 (0.95, 1.02)
Class 5	30	1.55 (0.35, 6.90)	0.79 (0.30, 2.12)	0.95 (0.29, 3.16)	0.16 (0.03, 0.77) *	1.02 (0.93, 1.12)
**Males 40 to 66**	**2,385**					
Class 1	81	0.13 (0.00, 28.12)	7.11 (3.67, 13.78) ***	0.87 (0.45, 1.68)	1.16 (0.79, 1.70)	1.03 (0.98, 1.09)
Class 2	738					
Class 3	1,173	1.70 (1.10, 2.61) *	0.69 (0.56, 0.85) **	0.87 (0.70, 1.08)	0.99 (0.86, 1.14)	1.02 (0.99, 1.04)
Class 4	293	2.20 (1.23, 3.92) *	0.52 (0.37, 0.72) **	0.73 (0.53, 1.01)	0.43 (0.34, 0.54) ***	0.99 (0.95, 1.02)
Class 5	100	2.13 (0.93, 4.86)	0.22 (0.12, 0.39) ***	0.60 (0.32, 1.12)	0.73 (0.52, 1.02)	1.05 (1.00, 1.10)

* p<0.05

** p<0.001

***p<0.0001

† Baseline BMI status as a three category variable (underweight, normal weight [referent], overweight/obese), and a dichotomous baseline urban-rural variable (referent: urban), age is scaled by a factor of 10 such that OR's represent change in odds with each decade increase in age, and year of study entry as a continuous variable.

††Reference groups for weight change trajectories were chosen on the basis of a pattern indicating “minimal weight change.”

In general, baseline study year was not significantly associated with trajectory membership. Notable exceptions include young males and young females (Table 3). For males 18 to 30, study year was statistically significant for all trajectories except for the moderate gain (medium) pattern. Young males had lower odds of following an initial loss and maintenance pattern and higher odds of rapid gain relative to the maintenance and slight gain weight referent trajectory. In contrast, females 18 to 30 had lower odds of following a slow gain (medium) and lower odds of initial gain with loss (medium) relative to the maintenance referent as study year increased.

Baseline age was statistically associated with trajectory membership for the majority of weight change trajectory groups for the two older age subgroups females such that for each ten year increase in age the odds of being in a weight gain pattern are lower than the weight maintenance referent (Table 3). The opposite relationship was seen for young adult females such that for each ten year increase in age, the odds of following a moderate gain (low), slow gain (medium), and initial gain with loss (medium) are 1.6, 3.0 and 3.2 times the odds of the weight maintenance (referent) group respectively.

Baseline weight status was the most significant predictor of trajectory membership for males and females of all age groups (Table 3). In general, individuals underweight at baseline had higher odds of being in a weight gain trajectory than a weight maintenance (referent) category. Additionally, for all age-gender subgroups, those who were overweight or obese at baseline had higher odds of being in an initial loss with maintenance relative to the maintenance referent.

## Discussion

In this paper we constructed weight change trajectories for Chinese adults participating in the CHNS during the 18 year period from 1991 to 2009. Through use of latent class trajectory analysis, we produced trajectories corresponding to patterns of weight change across the life cycle and examined correlates of these trajectories. Similar weight change trajectories were derived for each age by gender analysis subgroup, and some unique patterns were identified in age-group analyses that may have been lost in overall analyses. Our paper contributes to research supporting the importance of age-group specific analyses when deriving latent class trajectories for weight change.

Weight change trajectories were less varied in the oldest baseline age group (40 to 66y) of women as their change was characterized by four classes, while the younger women required six classes. The younger women (baseline 18–30y and 30–40y) had a pattern of weight gain followed by weight loss that was not seen in the oldest age group. In contrast, weight change was classified in fewer classes in the younger versus older baseline age groups for men. The baseline 18–30y men were characterized by two groups with some degree of weight maintenance, a large group with a period of maintenance and slow weight gain and a much smaller group with maintenance of weight loss. For comparability with the other age groups, we elected to use the larger maintenance and slow weight gain trajectory as our referent. Men 18–30y also had one unique trajectory group characterized by rapid gain followed by later weight maintenance. Among the highest membership classes for all age by gender groups were groups classified by maintenance or slow gain pattern of weight change.

Across all age and gender groups, the strongest predictor of trajectory membership was baseline weight status. Individuals who were underweight at baseline had higher odds of following a ‘weight gain’ pattern, regardless of initial gain and rate of gain, as compared to the maintenance referent. In general, individuals who were overweight or obese at baseline had higher odds of following an ‘initial loss with maintenance’ pattern and lower odds of following a weight gain trajectory compared to the maintenance referent. However, groups having ‘initial loss with maintenance’, characterized as having negative change followed by stability, were relatively small (ranging from 2.7% to 8.1%, with highest proportion in the 18–30y cohort)]. It is possible that a small fraction of the younger cohort was influenced by different body image ideals, or that a small fraction of the individuals who were heavier at baseline intentionally lost weight and maintained this weight loss over time. Additionally, men in the baseline age 30 to 40y group who were overweight or obese at baseline had higher odds of a slow gain (low) weight change pattern than ‘maintenance.’

The obesity epidemic is an emerging problem in developing countries [1, 23]. Developing countries whose problems were predicted to relate to inadequate food supply due to insufficient cultivated land are now undergoing their own obesity epidemics [23]. Even though prevalence of overweight and obesity may be higher in developed countries, the increased population density in developing countries translates into larger numbers of individuals being affected by obesity [1]. Dietary composition is changing in low income countries such that individuals in these developing populations have access to diets of increasing energy density [23]. An increase in BMI and obesity-related diseases such as diabetes has been detected in other developing countries such as India [24, 25].

Latent class analysis is a statistical method that serves to categorize individuals from a heterogeneous population into homogenous subgroups which exhibit meaningful differences with respect to a particular variable of interest, weight change in this case [14, 26]. Additional research can then determine other characteristics these groups have in common in order to inform possible public health interventions and recommendations for future weight control programs for these populations. While latent class trajectory models have been used extensively in the mental health and drug abuse literature [27, 28], behavioral and developmental psychology [11, 12], and recently to characterize dietary patterns [29, 30], they have been less frequently used to characterize changes in weight across the life course, but these models have the potential to be quite useful in this setting.

Research that has utilized latent class trajectory modeling for identification of changes in weight and BMI over time have focused on overall analyses for a large age range and many have computed such analyses using self-reported height and weight data [14–17]. Furthermore, some these studies provide cohort sequential data and may only provide repeated data for a random sample of the initial analysis sample over time [16, 17]. The data from the China Health and Nutrition Survey used in this paper provided 18 years of longitudinal data for which anthropometrics were obtained by trained health professionals, and additionally collected for the same household members over time [3, 18]. China has had dramatic modernization which has occurred very quickly in both urban and rural areas [22, 31]. We found that living in a rural area at baseline was not protective for weight change as originally hypothesized. In addition, age-group specific analyses are critical in order to identify unique growth differences during different life stages. The identified weight change trajectories discussed in this manuscript could be used in future analyses to determine if particular patterns of weight change are associated with increased risk of chronic disease and associated risk factors. Detection of high risk subgroups provides useful information to help target public health programs to certain groups. Furthermore, through conducting age-group specific analyses, these methods can better inform public program implementation to address important transitional time points for these high risk groups.

## Supporting Information

S1 Table Number (percent) of CHNS respondents with complete data by number of repeat visits: n (%)*, China Health and Nutrition Survey. *Some missingness by design due to exogenous weather shocks and temporary missingness and subsequent return of villages, with replacement enrollment of new villages with identical sampling techniques.
(DOCX)

S2 Table Prevalence of Overweight/Obesity* at each CHNS exam based on Asian obesity cut points (BMI ≥ 23 kg/m^2^) % (SE), China Health and Nutrition Survey. * Overweight/obesity classified using the Asian cut point for overweight and obesity (BMI ≥ 23 kg/m^2^) [20].
(DOCX)
