# Supplementary material for: Characterizing Long-Term Patterns of Weight Change in China Using Latent Class Trajectory Modeling
Source: PLoS One. 2015 Feb 20;10(2):e0116190. doi: 10.1371/journal.pone.0116190 (PMC4336139; doi:10.1371/journal.pone.0116190)
Supplement: S1 Table — *Some missingness by design due to exogenous weather shocks and temporary missingness and subsequent return of villages, with replacement enrollment of new villages with identical sampling techniques. (DOCX) [file pone.0116190.s001.docx]

| Table S1: Number (percent) of CHNS respondents with complete data by number of repeat visits: n (%)*, China Health and Nutrition Survey | | | | | | |
| --- | --- | --- | --- | --- | --- | --- |
| Number of Visits | | | | | | |
|  | 2  visits | 3  visits | 4  visits | 5  visits | 6  visits | 7  visits |
| Baseline Age |  |  |  |  |  |  |
| **Males**  **n=6,199** | **1,882 (30.4)** | **1,244 (20.1)** | **1,046 (16.9)** | **867 (14.0)** | **643 (10.4)** | **517 (8.3)** |
| 18-30 years  n=2,225 | 803 (36.1) | 462 (20.8) | 343 (15.4) | 295 (13.3) | 205 (9.2) | 117 (5.3) |
| 30-40 years  n=1,571 | 333 (21.2) | 275 (17.5) | 265 (16.9) | 269 (17.1) | 226 (14.4) | 203 (12.9) |
| 40-66 years  n=2,403 | 746 (31.0) | 507 (21.1) | 438 (18.2) | 303 (12.6) | 212 (8.8) | 197 (8.2) |
|  |  |  |  |  |  |  |
| **Females**  n=6,412 | **1,931 (30.1)** | **1182 (18.4)** | **979 (15.3)** | **875 (13.7)** | **677 (10.6)** | **768 (12.0)** |
| 18-30 years  n=2,184 | 862 (39.5) | 407 (18.6) | 288 (13.2) | 258 (11.8) | 186 (8.5) | 183 (8.4) |
| 30-40 years  n=1,785 | 351 (19.7) | 281 (15.7) | 266 (14.9) | 276 (15.5) | 269 (15.1) | 342 (19.2) |
| 40-66 years  n=2,443 | 718 (29.4) | 494 (20.2) | 425 (17.4) | 341 (14.0) | 222 (9.1) | 243 (10.0) |
|  |  |  |  |  |  |  |
| **Total Sample**  **n=12,611** | **3,813 (30.2)** | **2,426 (19.2)** | **2,025 (16.1)** | **1,742 (13.2)** | **1,320 (10.5)** | **1,285 (10.2)** |
| ***Some missingness by design due to exogenous weather shocks and temporary missingness and subsequent return of villages, with replacement enrollment of new villages with identical sampling techniques** | | | | | | |
