# Supplementary material for: Characterizing Long-Term Patterns of Weight Change in China Using Latent Class Trajectory Modeling
Source: PLoS One. 2015 Feb 20;10(2):e0116190. doi: 10.1371/journal.pone.0116190 (PMC4336139; doi:10.1371/journal.pone.0116190)
Supplement: S2 Table — * Overweight/obesity classified using the Asian cut point for overweight and obesity (BMI ≥ 23 kg/m2) [20]. (DOCX) [file pone.0116190.s002.docx]

| Table S2: Prevalence of Overweight/Obesity* at each CHNS exam based on Asian obesity cut points (BMI≥23kg/m^2^) % (SE), China Health and Nutrition Survey | | | | | | | |
| --- | --- | --- | --- | --- | --- | --- | --- |
|  | Study Year | | | | | | |
|  | 1991 | 1993 | 1997 | 2000 | 2004 | 2006 | 2009 |
| **Baseline Age** |  |  |  |  |  |  |  |
| **Males** |  |  |  |  |  |  |  |
| 18-30 years | 12.51 (0.011) | 12.24 (0.011) | 15.09 (0.011) | 16.81 (0.011) | 18.81 (0.011) | 19.98 (0.012) | 18.48 (0.012) |
| 30-40 years | 25.43 (0.015) | 25.35 (0.015 | 29.34 (0.015) | 32.40 (0.015) | 32.68 (0.015) | 33.43 (0.015) | 34.23 (0.015) |
| 40-66 years | 27.59 (0.012) | 28.49 (0.012) | 32.24 (0.013) | 35.70 (0.013) | 37.57 (0.013) | 39.51 (0.014) | 39.92 (0.016) |
| **Total Males** | **22.37 (0.007)** | **22.43 (0.007)** | **25.47 (0.008)** | **28.24 (0.007)** | **29.92 (0.008)** | **31.16 (0.008)** | **30.39 (0.008)** |
|  |  |  |  |  |  |  |  |
| **Females** |  |  |  |  |  |  |  |
| 18-30 years | 19.04 (0.013) | 18.95 (0.012) | 19.81 (0.012) | 20.54 (0.012) | 20.46 (0.012) | 21.55 (0.012) | 21.19 (0.013) |
| 30-40 years | 31.31 (0.015) | 31.26 (0.015) | 32.36 (0.015) | 33.89 (0.013) | 34.96 (0.014) | 34.81 (0.013) | 34.76 (0.014) |
| 40-66 years | 40.29 (0.013) | 40.74 (0.013) | 42.27 (0.013) | 45.78 (0.013) | 47.50 (0.014) | 49.60 (0.014) | 49.34 (0.016) |
| **Total Females** | **31.61 (0.008)** | **31.37 (0.008)** | **32.39 (0.008)** | **34.14 (0.008)** | **34.95 (0.008)** | **35.59 (0.008)** | **34.83 (0.008)** |
|  |  |  |  |  |  |  |  |
| **Total Sample** | **27.14 (0.005)** | **27.06 (0.005)** | **29.00 (0.006)** | **31.31 (0.005)** | **32.52 (0.006)** | **33.48 (0.006)** | **32.68 (0.006)** |
| *Overweight/obesity classified using the Asian cut point for overweight and obesity (BMI≥23kg/m^2^) [20] | | | | | | | |
